# Risk stratification of deep vein thrombosis using caprini score among post-operative patients

**DOI:** 10.6026/973206300213359

**Published:** 2025-09-30

**Authors:** Robin Abraham, Mahalakshmi B., Siva Subramanian N.

**Affiliations:** 1 Department of Medical & Surgical Nursing, Nootan College of Nursing, Sankalchand Patel University, Visnagar, Gujarat, India; 2 Department of Pediatric Nursing, Nootan College of Nursing, Sankalchand Patel University, Visnagar, Gujarat, India; 3 Department of Psychiatric Nursing, Nootan College of Nursing, Sankalchand Patel University, Visnagar, Gujarat, India

**Keywords:** Deep vein thrombosis, Caprini score, risk assessment, post-operative patients, critical care, clinical predictors, stratification

## Abstract

The analysis of risk stratification of deep vein thrombosis (DVT) among 120 post-operative patients in critical care units using the Modified Caprini Risk Assessment Tool is of interest. Most participants (over 80%) in both experimental and control groups were categorized as high risk for DVT. Statistical analysis revealed significant associations between higher Caprini scores and clinical variables such as age, BMI, mobility limitation, emergency surgery, central venous catheter presence, swollen leg, and longer surgical duration (p < 0.05). Demographic variables like gender, income, and dietary habits showed no significant association. Thus, we show the Caprini score as an effective tool for identifying high-risk patients who may benefit from early intervention. This study underscores the importance of pre-operative risk profiling to guide targeted DVT prevention strategies in critical care settings.

## Background:

Venous thromboembolism (VTE), which includes both deep vein thrombosis (DVT) and pulmonary embolism (PE), is a major cause of morbidity and mortality in hospitalized surgical patients [1]. Post-operative individuals, particularly those admitted to critical care units, are at significantly increased risk due to factors such as immobility, surgical trauma, inflammation, and the presence of comorbidities. Despite widespread awareness, DVT remains underdiagnosed and undertreated, often resulting in preventable complications such as fatal PE or chronic post-thrombotic syndrome [2]. To mitigate this risk, clinical guidelines emphasize early risk identification and thromboprophylaxis. One of the most widely validated tools for risk stratification is the Caprini Risk Assessment Model (RAM). This model calculates a cumulative score based on multiple risk factors, including age, body mass index, comorbidities, type and duration of surgery, and history of thromboembolic events. Higher Caprini scores correlate with increased DVT risk, guiding clinicians in tailoring prophylactic strategies [3]. Several studies have demonstrated the predictive accuracy of the Caprini Risk Assessment Score in identifying patients at increased risk for venous thromboembolism (VTE), particularly in surgical and hospitalized populations. It has been validated across diverse clinical settings and is widely used to guide prophylactic strategies, ensuring timely and individualized patient careSimilarly, in critically ill surgical patients, Caprini scores greater than 8 were associated with an 11.5% DVT incidence, compared to just 3.5% in low-risk patients [4]. Further evidence supports the Caprini model's relevance across surgical specialties. In laparoscopic colorectal cancer surgeries, Caprini scores showed significant correlation with DVT risk, with a sensitivity of 80.6% at a cutoff of 10.5 [5]. In plastic and reconstructive surgery, patients with scores >8 had nearly 20 times higher odds of developing post-operative DVT [6]. In surgical intensive care settings, substratification within the highest-risk Caprini group (score ≥5) has shown further refinement in predicting thromboembolic events. For example, among cancer patients admitted post-operatively to ICU, those with scores >10 had significantly higher odds of VTE than those scoring 5-6 or 7-8 [7]. These findings are crucial because nearly all ICU patients post major surgery already fall into the "high-risk" category, limiting the discrimination power of the original tool without further stratification. However, some limitations remain. Not all populations may fit the model equally well. For instance, in orthopedic patients, the Caprini score underperformed compared to alternative tools like the Padua score [8]. Therefore, it is of interest to assess and describe the utility of the Caprini Risk Assessment Score in critically ill post-operative patients in intensive care, with a focus on identifying DVT risk patterns and refining thresholds for better stratification and targeted prophylaxis.

## Methodology:

## Research design:

This study utilized a descriptive cross-sectional research design with comparative analysis to assess the risk of deep vein thrombosis (DVT) among post-operative patients in critical care units and evaluate the effectiveness of prophylactic measures based on risk stratification.

## Study setting and population:

The research was conducted in the critical care units of Welfare Hospital and Kaka Ba Hospital, Bharuch, Gujarat. The population comprised post-operative patients aged above 41 years, who were admitted to the ICU following elective or emergency surgeries. All patients were medically stable and conscious, and those with pre-existing DVT were excluded.

## Sampling technique and sample size:

A total of 120 post-operative patients were selected through convenience sampling.

## Data collection tools and interventions:

Structured questionnaire to assess demographic information and Modified Caprini Risk Assessment Scale was used to collect data.

## Data analysis:

Data were analyzed using descriptive statistics including frequency, percentage, mean, and standard deviation to summarize patient demographics, clinical variables, and DVT risk levels.

## Results:

Table 1 (see PDF) presents the baseline distribution of DVT risk among post-operative patients using the Caprini score, showing that out of 120 participants, 97 (80.8%) were categorized as high risk (score ≥5), while only 23 (19.2%) were in the moderate risk group (score 3-4). This highlights a predominantly high-risk surgical population requiring vigilant thromboprophylaxis. Table 2 (see PDF) examines the association between Caprini scores and various demographic and clinical variables. Significant associations were observed with age (χ^2^=32.427, p<0.001), occupation (χ^2^=17.342, p<0.001), BMI (χ^2^=18.941, p<0.001), nature of surgery (χ^2^=5.046, p=0.025), mobility (χ^2^=30.788, p<0.001), central venous catheter presence (χ^2^=9.288, p=0.002), swollen leg (χ^2^=21.497, p<0.001), and duration of surgery (χ^2^=12.564, p=0.006). These results indicate that older age, higher BMI, reduced mobility, use of central venous catheters, and prolonged surgeries are significant predictors of elevated DVT risk. Conversely, variables such as gender, income, habits, and medical history were not significantly associated with Caprini scores.

## Discussion:

The present study evaluated deep vein thrombosis (DVT) risk among post-operative patients in critical care using the Modified Caprini Risk Assessment Model. It revealed that 80.8% of patients were categorized as high risk (score ≥5), demonstrating a strong need for targeted thromboprophylaxis in this vulnerable population. The significant associations identified between higher Caprini scores and clinical variables such as age, BMI, limited mobility, emergency surgery, central venous catheter use, swollen leg, and longer surgery duration underscore the tool's clinical relevance. These findings align with multiple large-scale validations of the Caprini score, including studies by Bo *et al.* (2016) [9] which observed a 16-fold increase in DVT risk in patients with scores ≥9, and Obi *et al.* (2015) [4] who reported DVT incidence of 11.5% in high-risk ICU patients compared to just 3.5% in lower-risk groups. Numerous other studies confirm the reliability of the Caprini score in stratifying surgical and ICU patients. For example, Krishnamoorthy *et al.* (2024) found a significant rise in DVT incidence in patients with scores above 7, which closely mirrors our results [10]. (Lobastov *et al.* 2016) reported that patients with Caprini scores over 12 had a DVT incidence as high as 65% [11]. In elderly women undergoing colpocleisis, Wang *et al.* (2024) also found a significant correlation between Caprini scores ≥7 and DVT development. Among burn patients, Xie *et al.* (2025) [12] reported strong predictive power of the Caprini model with an AUC of 0.853. In orthopedics, (Dashe *et al.* 2019) [13] validated the score's predictive power for DVT post-surgery, while Anand *et al.* (2023) [8] showed that the Caprini model outperformed the Padua score in predicting thrombotic risk in surgical populations. Regarding age emerged as a highly significant predictor (p<0.001), with older patients tending to score higher on the Caprini scale. This aligns with multiple studies, including those by Bo *et al.* (2016) and Wang *et al.* (2025) [14] which reported significantly increased DVT incidence in elderly post-operative patients. Body Mass Index (BMI) also showed a strong positive association with higher Caprini scores (p<0.001), which is consistent with prior findings. Obesity contributes to increased intra-abdominal pressure and venous stasis, both of which are known risk factors for DVT. Similar associations were observed in studies by Lobastov *et al.* 2024) [11], who found that obese patients consistently scored higher and were more likely to develop DVT post-operatively. Mobility limitation factor was another key variable significantly associated with high Caprini scores (p<0.001). Reduced mobility, particularly in ICU settings, leads to venous stasis, which directly contributes to thrombus formation. This relationship has been well-documented in studies like Jeong *et al.* (2014) and Ajdarpasic & Hawari (2024), [15, 16], both of which emphasized that immobility significantly elevates DVT risk, particularly in the post-operative recovery phase. The presence of a central venous catheter (CVC) was significantly linked to higher Caprini scores (p=0.002), supporting previous research that CVCs can act as a source of endothelial injury and flow disturbance, promoting clot formation. This is consistent with findings from Fu *et al.* (2018) [17], who recommended integrating catheter-related factors into more refined thrombosis risk models. Swollen leg as a clinical sign was also strongly associated with elevated scores (p<0.001), which is not surprising as it reflects potential early signs of thrombosis. Swelling is a classic symptom of lower-limb DVT and serves both as a scoring criterion and a red flag in clinical assessment, as supported by Lu *et al.* (2021) [5]. Duration of surgery was another significant factor (p=0.006), with longer procedures correlating with higher Caprini scores. Extended surgery time increases the period of immobility and endothelial exposure, both of which contribute to thrombogenesis. This has been observed across specialties, particularly in colorectal and orthopedic surgeries, as demonstrated by [8]. Additional research across varied surgical populations further validates the Caprini score's predictive utility for deep vein thrombosis (DVT). In elderly women undergoing colpocleisis, a Caprini score ≥7 was significantly associated with postoperative DVT, with the model achieving a sensitivity of 77.3% and an AUC of 0.758 [18]. In laparoscopic colorectal cancer surgery, Zhang *et al.* found that patients with Caprini scores ≥10 and D-dimer >0.835 µg/mL had a DVT incidence of nearly 39%, suggesting that dual-factor assessment enhances risk precision [19]. Among burn patients, higher Caprini scores corresponded to a markedly elevated DVT risk, and a predictive model using the score achieved an AUC of 0.853, confirming its strong discriminatory power. In orthopedic surgery, combining Caprini scores with ultrasound parameters significantly improved prediction accuracy for lower extremity DVT, with combined AUC values exceeding 0.95 [20]. A separate study found that patients with Caprini scores >8 had significantly higher DVT incidence, and thromboprophylaxis in this group was highly effective at reducing events [21]. Lastly, a comparative study confirmed the Caprini score's superior predictive power over the Padua model in surgical patients, explaining a greater proportion of DVT risk variability [22]. Interestingly, demographic variables such as gender, income, dietary habits, and medical history were not significantly associated with Caprini scores in this study. This finding is consistent with several prior investigations where such factors, while potentially influencing overall health, did not show a direct relationship with thrombosis risk in post-operative settings [23, 24]. This underscores the strength of the Caprini model in focusing on pathophysiological and procedural variables rather than broader sociodemographic factors.

## Conclusion:

Caprini score's sensitivity to clinically meaningful predictors of DVT such as advanced age, obesity, immobility, invasive devices, and surgery complexity. These variables should be prioritized in risk assessment protocols, while less predictive demographic traits can be deprioritized in clinical decision-making. This reinforces the Caprini model's practicality and specificity in high-stakes environments like the ICU. The study confirms the Caprini score as an effective tool for identifying high-risk DVT patients in critical care. Significant clinical factors like age, BMI, mobility, and surgery duration were strongly associated with higher scores. Non-clinical variables showed no impact. Routine Caprini-based risk assessment can improve targeted DVT prevention.
